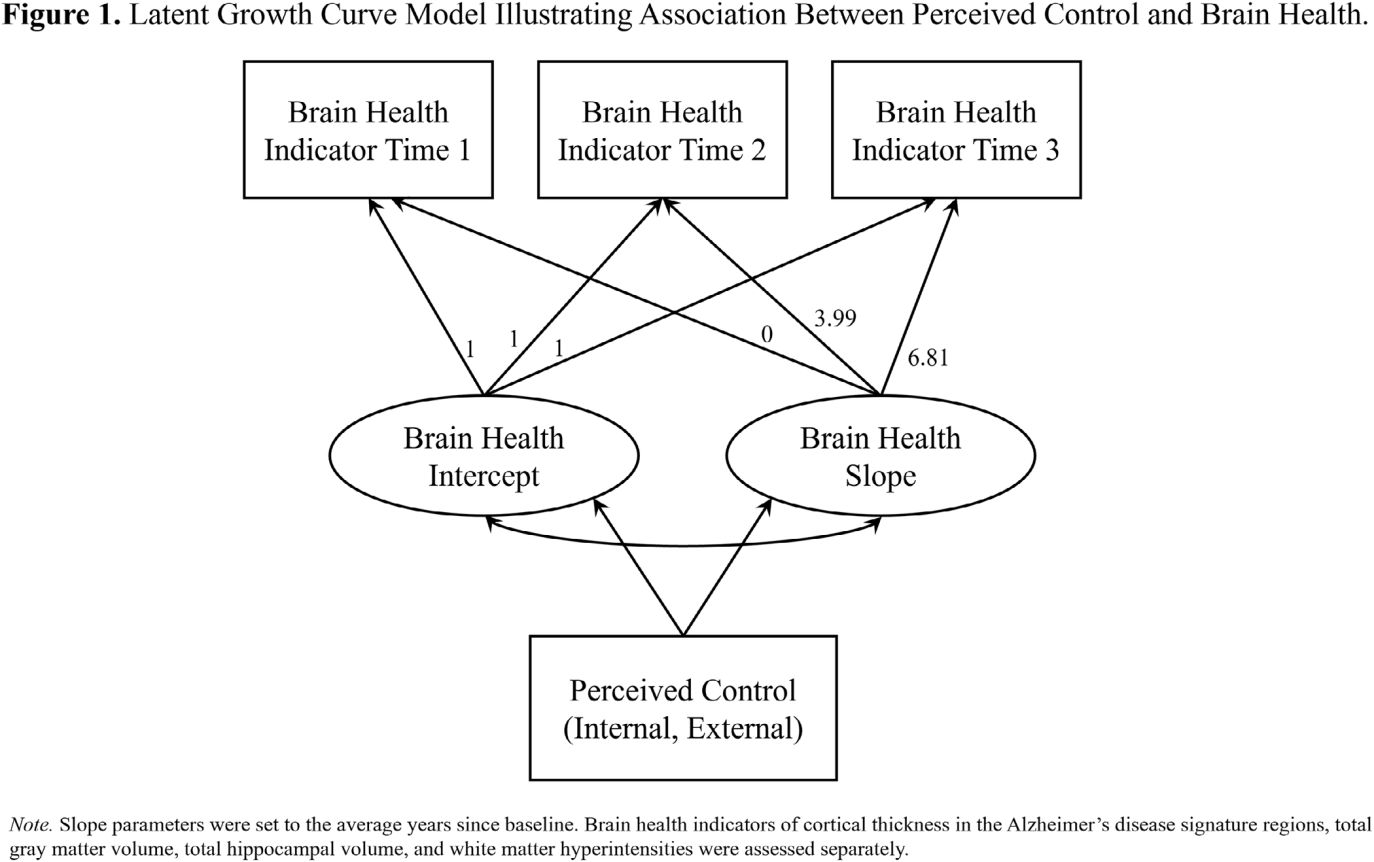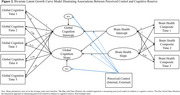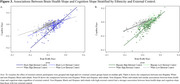# Perceived Control, Brain Health, and Cognitive Reserve: Assessing Longitudinal Resilience Mechanisms in Diverse Older Adults

**DOI:** 10.1002/alz70860_097207

**Published:** 2025-12-23

**Authors:** Monica E. Walters, Emily P. Morris, Jordan D. Palms, Robrielle M. Pierce, Kiana A. Scambray, Ketlyne Sol, Adam Brickman, Richard Mayeux, Laura B. Zahodne

**Affiliations:** ^1^ University of Michigan, Ann Arbor, MI, USA; ^2^ Columbia University Irving Medical Center, New York, NY, USA

## Abstract

**Background:**

Perceived control is a psychosocial factor consisting of internal and external control. Although higher internal and lower external control relate to better cognition, mechanisms underlying these associations are unclear. We assessed whether internal and external perceived control related to two longitudinal resilience mechanisms, brain maintenance and cognitive reserve, across a diverse sample of older adults.

**Methods:**

Participants included non‐Hispanic White (*n* = 199), non‐Hispanic Black (*n* = 262), and Caribbean Hispanic (*n* = 319) older adults (*M_age_* = 74.48, *SD* = 6.04) from the Washington‐Heights/Inwood Columbia Aging Project. Brain health included cortical thickness in the Alzheimer's disease signature regions, total hippocampal volume, total gray matter volume, white matter hyperintensities, and their composite. Global cognition was a composite of processing speed, language, memory, and visuospatial functioning. Internal and external control were separately assessed in relation to (a) longitudinal markers of brain health and (b) longitudinal cognitive reserve, both as a predictor of global cognition trajectories while controlling for brain health (residual approach) and as a moderator of associations between longitudinal brain health and global cognition (interaction approach). Univariate and bivariate latent growth curve models and multiple‐group models were used. Covariates included age, sex/gender, years of education, and intracranial volume.

**Results:**

Greater internal control related to greater baseline cortical thickness for individuals of Caribbean Hispanic ancestry only. Greater external control related to greater baseline hippocampal volume for non‐Hispanic Black individuals, but faster decline in hippocampal volume for non‐Hispanic White individuals. Controlling for brain health, greater external control related to worse cognition initially and over time for the entire group. Additionally, greater decline in brain health more strongly related to greater decline in cognition at higher levels of external control. This effect was driven by Hispanic and non‐Hispanic Black individuals.

**Conclusions:**

We found more evidence for cognitive reserve, rather than brain maintenance, as a resilience mechanism linking perceived control to cognitive health. Internal and external control have distinct associations with brain and cognitive health across ethnic groups, highlighting the importance of understanding upstream factors contributing to perceived control differences. Reducing external control, especially among minoritized older adults, may result in better cognitive health through a cognitive reserve mechanism.